# Perfusion MRI for monitoring therapy effects in experimental chronic limb ischemia

**DOI:** 10.1186/1532-429X-11-S1-P136

**Published:** 2009-01-28

**Authors:** Harald Kramer, Steven Sourbron, Rabea Hinkel, Franziska Globisch, Christian Kupatt-Jeremias, Maximilian F Reiser, Bernd J Wintersperger

**Affiliations:** University Hospital of Munich, Munich, Germany

**Keywords:** Limb Ischemia, Arterial Input Function, Extraction Flow, Therapy Effect, Spatial Resolution Imaging

## Introduction

Until today, imaging of lower extremity blood supply and perfusion is limited to the display of the macroscopic vasculature by angiographic methods like DSA, CTA or MRA. However, all these methods are limited to high spatial resolution imaging of the arterial or venous vessels but do not display changes at the microvascular level in regard to tissue perfusion. MR perfusion imaging may overcome this limitation in experimental and clinical settings and may allow non-invasive monitoring of modern therapeutic options including angiogenisis.

## Purpose

To implement and evaluate the use of perfusion MR in monitoring angiogenetic therapies and their effects based on a rabbit model of iatrogenic induced chronic lower limb ischemia.

## Methods

**Figure 1:**
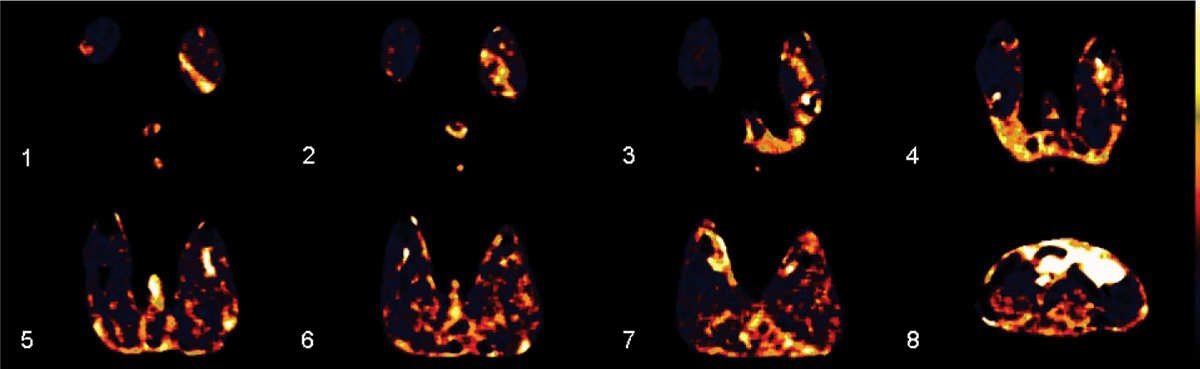
**Colour coded plasma flow images over 8 slices (1 = distal, 8 = central) at day 7 after left side SFA excision**. Images show significantly higher plasma flow on the ischemic side before angiogenetic therapy.

MR perfusion imaging was performed in 8 rabbits with chronic lower limb ischemia after unilateral SFA excision at 3 Tesla (Magnetom Verio, Siemens Healthcare) using a 32-element coil. In 4 animals MRI was performed at day 7 and in 4 animals at day 7 and 35 (after angiogenesis therapy). Multi-slice coverage was provided using a 2D-TurboFLASH technique at 1.5 s temporal resolution with repeated measurements over 10 min after injection of 0.1 mmol/kg gadobutrol (Gadovist, Bayer Schering Pharma). One slice was placed through the aorta for measuring the Arterial Input Function (AIF) and 7 sliced covered the gluteal, thigh and knee musculature. Precontrast T1-mapping was performed using a variable TI GRE technique at identical slice locations (27 steps from 110 ms – 5 s). Data were post-processed off-line using in-house written software PMI 0.3 including calculation of plasma flow (PF), plasma volume (PV) and extraction flow (EF) based on a 2-compartmental model. T1 mapping data were used to convert signal-time courses to tracer concentration. Figure 1.

## Results

T1-maps produced robust results on with precontrast T1-values close to typical reference values at 3 T. PF was significantly different between the non-ischemic and ischemic limb (14.3 ± 11.5 vs. 8.4 ± 3.9 ml/100 ml/min) on day 7 whereas there was no significant difference on day 35 after therapy (9.3 ± 2.3 vs. 10.5 ± 3.2 ml/100 ml/min). EF showed similar findings with 2.17 ± 1.71 vs. 1.71 ± 1.6 ml/100 ml/min on day 7 and 2.3 ± 1.98 vs. 2.15 ± 1.68 ml/100 ml/min on day 35. PV did not show significant differences either before or after therapy.

## Conclusion

Chronic experimental limb ischemia results in significant changes of MR derived perfusion parameters, with reconstitution after experimental therapy. Initial data indicate that perfusion MRI provides a useful tool for the evaluation of muscle ischemia and of therapy effects.

